# Effective Connectivity in Response to Posture Changes in Elderly Subjects as Assessed Using Functional Near-Infrared Spectroscopy

**DOI:** 10.3389/fnhum.2018.00098

**Published:** 2018-03-16

**Authors:** Congcong Huo, Ming Zhang, Lingguo Bu, Gongcheng Xu, Ying Liu, Zengyong Li, Lingling Sun

**Affiliations:** ^1^Key Laboratory of High Efficiency and Clean Mechanical Manufacture, School of Mechanical Engineering, Shandong University, Jinan, China; ^2^Department of Biomedical Engineering, Faculty of Engineering, The Hong Kong Polytechnic University, Hong Kong, Hong Kong; ^3^Beijing Key Laboratory of Rehabilitation Technical Aids for Old-Age Disability, National Research Center for Rehabilitation Technical Aids Beijing, Beijing, China; ^4^Key Laboratory of Rehabilitation Aids Technology and System of the Ministry of Civil Affairs, Beijing, China

**Keywords:** posture change, aging, effective connectivity, near-infrared spectroscopy, dynamic Bayesian inference

## Abstract

This study aims to assess the posture-related changes in frequency-specific effective connectivity (EC) in elderly subjects by coupling function measured using functional near-infrared spectroscopy (fNIRS). The fNIRS signals were continuously recorded from the bilateral prefrontal cortex (PFC), motor cortex (MC), and occipital lobe (OL) in 17 healthy elderly and 19 healthy young subjects during sitting and standing states. EC was calculated based on Dynamic Bayesian inference in one low frequency interval I: 0.052–0.145 Hz and one very low frequency interval II: 0.021–0.052 Hz. Results show that in response to posture change, the coupling strength significantly increased in interval I of the young group from right PFC to MC (*p* < 0.05). Meanwhile, the coupling strength of the elderly group was significantly increased in interval II from the left PFC to right PFC (*p* = 0.008) and to left MC (*p* = 0.031) in the standing state as compared with that in the sitting state. Compared with that of the young group, the coupling strength of the elderly group was significantly decreased (*p* < 0.05) between the right PFC and left PFC in interval I and from PFC and OL to MC in interval II during the sitting state. The decreased EC in interval I was also positively correlated with cognitive scores in the elderly group. In addition, the coupling strength from MC to PFC in interval II during standing state was significantly increased in elderly subjects as compared with that in the young group. These results revealed the age-related changes in reorganization of interregional interactions for different postures. These findings may provide evidence of impaired cognitive function in the elderly and can deepen the understanding on age-related changes in neurovascular coupling.

## Introduction

Normal aging is related with decreased cognitive functions, including lack of attention, memory loss, and executive function decline (Hedden and Gabrieli, 2004; Kiyoka et al., 2013), which seriously affect their quality of life (Mitchell et al., 2010). Age-related cognitive decline is accompanied by the changes in neuronal activity and attenuation of functional interaction among different brain regions (Taniwaki et al., 2007; Spreng and Schacter, 2012). Maintaining a static standing posture is essential in executing daily activities. Analyzing of the age-related changes in brain network connections through different body postures allows an in-depth detection of the functional performance of neurovascular coupling mechanism of elderly people. However, the age-related changes in the reorganization of directed interregional interactions on the brain network under different posture states remain poorly understood.

Brain vasculature can respond to different conditions to regulate the blood flow. Regional cerebral blood flow (rCBF) must be allocated appropriately according to compartmentalized brain functions to meet different functional requirements. This process involves neurovascular coupling (Hotta, 2016). From sitting to standing states, the cardiovascular system maintains a stable rCBF to support sufficient levels of brain activity through myogenic, neurogenic, or metabolic mechanisms (Beek et al., 2008). These physiological mechanisms play a role in neurovascular coupling. Age-related functional deterioration of neurovascular coupling (Stefanova et al., 2013; Fabiani et al., 2014) contributes to the decline in high cortical functions, including cognition (Sorond et al., 2013) and motor coordination (Sorond et al., 2011). Hence, age-related deterioration may be reflected in the brain functional network based on neurovascular coupling. New strategies must be developed to determine the prognosis and monitor the cognitive decline with aging. This study on posture-dependent brain network provides an in-depth understanding of the age-related changes in neurovascular coupling.

Functional near-infrared spectroscopy (fNIRS) is a non-invasive neuroimaging technology that is particularly sensitive to microvasculature (Obrig et al., 2000; Li et al., 2010; Sasai et al., 2012). The cerebral signals measured by fNIRS consist of endogenous systemic activity and the neurovascular coupling (Scholkmann et al., 2014). This method is used to monitor brain activities by measuring the relative regional changes in hemoglobin concentration and analyzing tight neurovascular coupling between neural activity and rCBF (Girouard and Iadecola, 2006; Kamran et al., 2015). Moreover, fNIRS are currently used to acquire brain signals for brain machine interface (BCI) and have been popularized due to their safety and well-balanced spatial and temporal resolution (Naseer and Hong, 2015; Noori et al., 2017). Sasai reported that oscillation signals, which were simultaneously recorded from fNIRS and functional magnetic resonance imaging (fMRI), are well correlated (Sasai et al., 2012). In comparison with fMRI, fNIRS exhibited some advantages, such as relatively high temporal resolution (10 Hz) and insensitivity to movement artifacts. Hence, fNIRS is especially appropriate for examining brain function of the elderly in natural environments.

The brain is a complex network that includes multiple regions with specific functions. Information processing requires functional dynamic interaction among segregated brain areas (Sporns and Kötter, 2004). One method of characterizing the interaction relationship is functional connectivity (FC), which describes such relationship as “statistical dependencies among distinct neurophysiological regions” (Biswal et al., 2010; Friston, 2011). Reconstructing dynamic human movement from a sequence of static body postures could increase the FC that is related to the motor cortex (MC) (Orgs et al., 2016). Our previous study revealed posture-related changes in the brain FC based on wavelet phase coherence in elderly subjects (Wang et al., 2016). However, analysis of FC does not involve inference on coupling among brain regions. The interactions among different brain areas are dynamic and directional, but the results cannot reveal the causal interaction and direction of influences in the brain network. To fully understand the neurovascular coupling interaction among brain regions, we adopted effective connectivity (EC) to investigate these interactions (Park and Friston, 2013). EC refers explicitly to “the causal influence one neural system exerts over another” and can characterize the dynamic causality of the brain network (Friston, 1994). Granger causality is a popular method for calculating EC in fMRI. However, this method can only infer the existence of causal effect but not the causal coupling *per se* (Park and Friston, 2013). Hence, the EC calculated by Granger causality is regarded as a measure of directed FC. By comparing with Granger causality, we based the Bayesian inference on a dynamical model that can infer the underlying causal mechanisms of the brain (Friston, 2011).

In this study, the prefrontal cortex (PFC), MC, and occipital lobe (OL) were covered by 36 channels to measure oscillation signals with fNIRS. PFC is essential for emotional control and plays an important role in planning complex cognitive behavior, decision making, and forming personal expressions (Miller and Cohen, 2001). PFC may also be involved in maintaining attention-demanding balance tasks (Mihara et al., 2008; Basso et al., 2014). MC is mainly responsible for coordination of sensory and motor functions (Hogenhout, 2013). OL is crucial in coordination of language, perception, and abstraction and is essential for processing visual information (Moran and Desimone, 1985; Corbetta et al., 1991; Astafiev et al., 2004). These regions may be involved in a differently coordinated fashion in response to posture changes.

In this study, we hypothesized that age-related changes exist in EC network in response to posture change. The directed coupling interactions among the brain regions were calculated based on a mode of coupled phase oscillator and Dynamical Bayesian inference (Tomislav et al., 2016), from which the influence exerted by one oscillator on another was determined, and the underlying causality was predicted. Analyzing the age-related changes in brain network connections through in different posture states may deepen our understanding of the basis of function deficits, which may emerge during normal (non-pathological) aging. This study offers a new method of accessing and preventing cognitive loss and detecting the functional performance of neurovascular coupling in the elderly.

## Materials and methods

### Participants

Thirty-eight healthy volunteers were recruited from the university and categorized in two age groups: the first group (elderly group) was composed of 18 elderly subjects (all right-handed; 5 females, 13 males), the second group (young group) was composed of 20 healthy young subjects (all right-handed; 7 females, 13 males). The subjects were screened to ensure that they did not have a history of hypertension, abnormal heart, kidney, liver, and lung functions. In this study, hypertension was defined as systolic blood pressure ≥140 mm Hg or diastolic blood pressure ≥90 mm Hg (Jones et al., 2003). All subjects were free from psychiatric disorders or neurological illness and were not treated with any psychotropic medication. All participants provided written informed consent before participating in this study. The experimental procedure was approved by the Human Ethics Committee of National Research Center for Rehabilitation Technical Aids and was in accordance with the ethical standards specified by the Helsinki Declaration of 1975 (revised in 2008).

### Data acquisition

**Figure 1:**
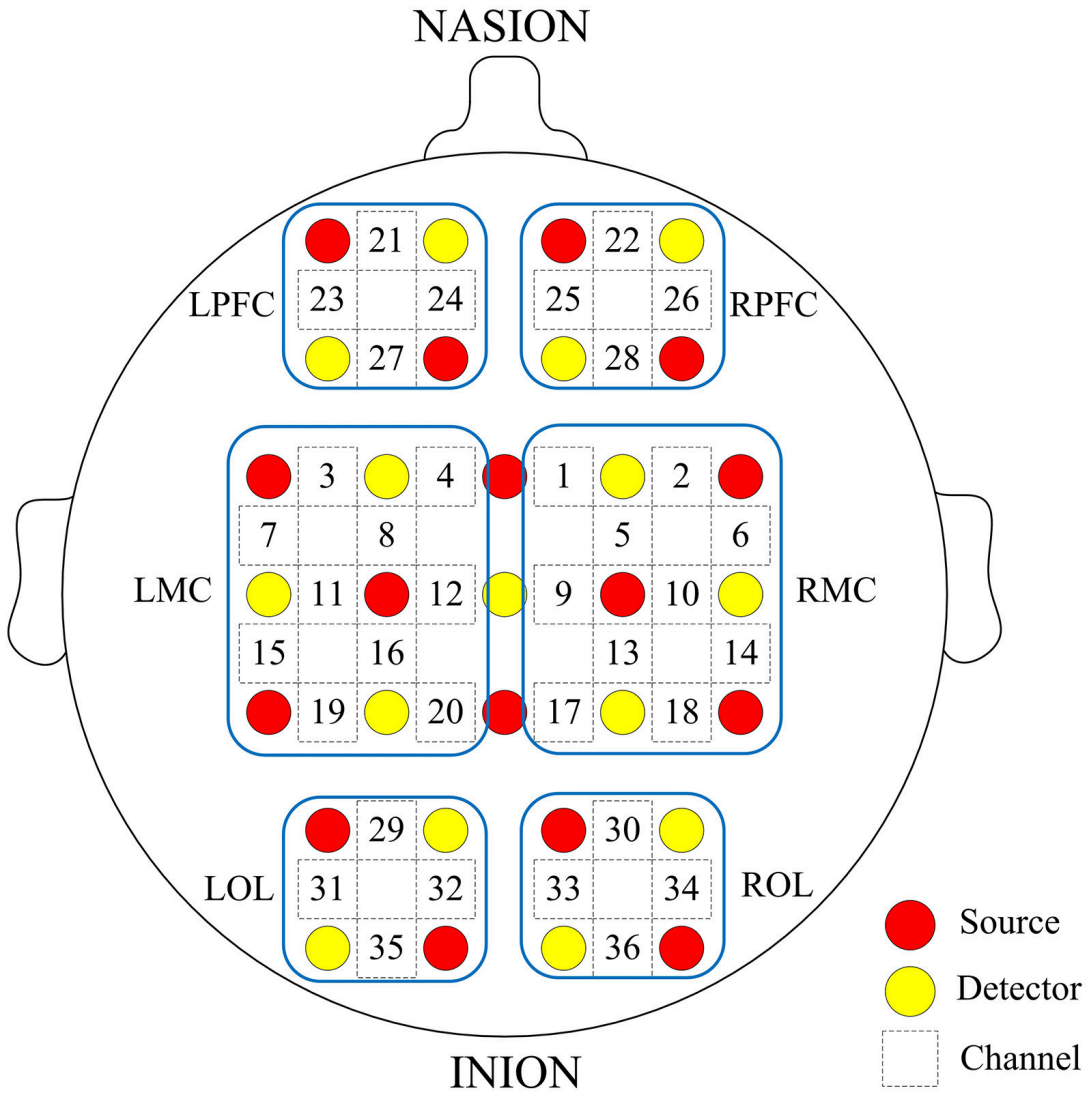
Arrangement of source optodes (red dots), detector optodes (yellow dots), and measurement channels (white rectangle) in the prefrontal cortex, motor cortical and occipital lobe based on the international 10/10 system. Six cerebral cortex areas are separated by the blue frames as LPFC, RPFC, LMC, RMC, LOL, and ROL.

Prior to the experiment, we recorded basic information of our subjects, including age, sex, height, weight, and their individual the Mini Mental State Examination (MMSE) scores. Table 1 shows the characteristics of the participants. fNIRS measurements were conducted with a multi-channel tissue oxygenation monitor (NirScan Danyang Huichuang Medical Equipment CO. Ltd) with three wavelengths (755, 808, and 855 nm) to measure the change in regional blood volume. The reflection of the infrared light was tested by source probes placed on the scalp 30 mm away from the detector probes. We initially set all differential path-length factors to 6.0. Tissue absorbency measurements were recorded and converted into optical density units, and the concentration changes in HbO2 (Delta [O2Hb]) were calculated based on the modified Beer-Lambert law (Teng et al., 2006). The sampling rate was 10 Hz. Recordings were derived from 36 channels contributed by sources and detectors (Figure 1), which were positioned over the left PFC (LPFC), right PFC (RPFC), left MC (LMC), right MC (RMC), left OL (LOL), and right OL (ROL) in accordance with the International 10/10 System. Each channel arrangement was accurately corresponded to the 10/10 electrode positions according to different head sizes by using the calibration function of the instrument and the corresponding template.

**Table 1:** Demographic information.

**Parameter**	**Old (*n* = 17)**	**Young (*n* = 19)**	***p* for difference**
Age (years)	69.4 (9.6)	24.1 (1.9)	0.000^*^
BMI	19.2 (3.2)	18.5 (4.2)	0.584
MMSE	27.5 (1.5)	29.3 (0.6)	0.000^*^

*Values are presented as means and standard deviations. BMI, Body Mass Index; MMSE, Mini-mental state examination*.

* *Significant group differences p < 0.05*.

The probes were covered with a loose-fitting black cloth to minimize interference of ambient light on optical signals. Measurements were performed in a quiet room with dim light at room temperature. The subjects were instructed to maintain a comfortable sitting position, remain still, relax with eyes closed, and avoid drastic movements. Signal recordings were collected at the sitting state for 15 min. The subjects were reminded to stay awake and not fall asleep. After collecting the sitting state data, we required the subjects to stand up and maintain their balance. Signal recordings were collected at the standing state for 10 min. After completion of the experiment, a questionnaire survey was conducted to examine whether or not the subjects were asleep during resting state, and to ensure quality of the experiment.

### Data pre-processing

The pre-processing method for fNIRS data, which has been described in detail in previous studies (Han et al., 2014; Xu et al., 2017), was conducted as follows. First, moving average method was applied to remove the noise-like abrupt spikes caused by movement artifacts or background light. This method was based on moving standard and spine interpolation routines. Second, to remove uncorrelated noise components (above 2 Hz) and low-frequency variations (below 0.005 Hz), we employed a Butterworth band-pass filter to obtain filtered signals of 0.005–2 Hz. One young subject and one elderly subject were excluded because of excessive head artifact interference in their signals.

### Wavelet transform (WT)

WT is a method that transforms signals from the time domain to the time–frequency domain by using frequency-adjustable filtering window and obtains a three-dimensional map containing time–frequency–amplitude information. The method of WT has been described previously in detail (Han et al., 2014). At a specific frequency *f* and time point *t*_*n*_, complex wavelet coefficients obtained by WT are defined as:

(1) $$w_{k}\left(t_{n}\right)=W_{k}\left(f,t_{n}\right)\cdot e^{i\;\emptyset_{k}\left(f,t_{n}\right)}=a_{k}\left(f,t_{n}\right)*ib_{k}\left(f,t_{n}\right)$$

Wavelet instantaneous phase is defined as Φ_*k*_(*f, t*_*n*_):

(2) $$\Phi_{k}(f,t_{n})=\arctan(b_{k}(f,t_{n})/a_{k}(f,t_{n}))$$

Through WT, the phase information was extracted from each filtered time series to build a series of coupled-phase-oscillator models.

These oscillation signals measured by fNIRS implied different physiological meanings at different frequency intervals, reflecting myogenic, neurogenic, or metabolic regulation of microvascular blood flow (Han et al., 2014; Vermeij et al., 2014). Two major frequency-specific oscillator intervals have been distinguished by WT in the intervals as follows: I-myogenic activity (0.052–0.145 Hz) and II-neurogenic activity (0.021–0.052 Hz) (Shiogai et al., 2010).

### Coupling strength and direction

Bayesian theory is based on prior knowledge used to estimate current or posterior model parameters or uses the acquired knowledge to improve the reasoning (Stankovski et al., 2014).

(3) $$p_{\chi }\left(M|\chi \right)=\frac{\zeta \left(\chi |M\right)p_{prior}(M)}{\int \zeta \left(\chi |M\right)p_{prior}\left(M\right)dM}$$

The properties of a network of N-coupled periodic oscillators can be analyzed based on its phase dynamics. A system of N stochastic differential equations varying with time was established according to the original signal phase information obtained by WT (Stankovski et al., 2014):

(4) $$\Phi_{k}\left(t\right)=w_{k}\left(t\right)*q_{k}\left(\Phi_{1},\Phi_{2},\ldots ,\Phi_{n},t\right)*\xi_{k}(t)$$

Where *k* = 1, ⋯, *N*. *N* is the number of oscillators in the network; in this paper, *N* = 2; ω_*k*_(*t*) is defined as the parameter for the natural frequency; the deterministic base function *q*_*k*_ describes the interacting dynamics of the coupled phase oscillators Φ_1…*N*_; and ξ_*k*_ is considered as Gaussian white noise. The deterministic periodic part of the differential equation (4) for each oscillator is decomposed into a sum of base functions Φ_*k*_ = exp[*l*(*k*_1_φ_1_ + *k*_2_φ_2_ + … + *k*_*N*_φ_*N*_)] modulated by parameter $c_{b}^{\left(k\right)}$ varying with time by using Fourier approximation (Stankovski et al., 2014):

(5) $${\dot{\Phi }}_{k}(t)=\sum_{b\;=\;-B}^{B}{c_{b}}^{\left(k\right)}\phi_{b}(\Phi_{1},\Phi_{2},\cdot \cdot \cdot ,\Phi_{n})*\xi_{k}(t)$$

The deterministic part $c_{b}^{\left(k\right)}$ is decomposed into the sum of the partial contributions of different orders of coupling to isolate the different orders of the network coupling:

(6) $$\begin{array}{l} {\dot{\Phi }}_{k}(t)=c_{0}^{\left(k\right)}*\sum_{b=-B}^{B}c_{b}{}^{\prime {\left(k:l\right)}_{\phi_{b}(\Phi_{l})}}*\sum_{b=-B}^{B}c_{b}{}^{\prime \prime {\left(k:l,m\right)}_{\phi_{b}(\Phi_{l},\Phi_{m})}}\\ \;*\sum_{b=-B}^{B}c_{b}{}^{\prime \prime \prime {\left(k:l,m,n\right)}_{\phi_{b}(\Phi_{l},\Phi_{m},\Phi_{n})}*\cdot \cdot \cdot *\xi_{k}(t)} \end{array}$$

Where *k* ≠ 0; *l, m, n* = 1, … *N* and *l nem nen*; $c_{b}^{\left(k\right)}$the vector of coefficients of different orders of the coupling. The model's Fourier components act as base function for the Dynamical Bayesian inference and were used to evaluate the parameters $c_{b}^{\left(k\right)}$. We recursively calculated the parameters $c_{b}^{\left(k\right)}$ using the equations listed in the paper (Stankovski et al., 2014) based on Bayesian theory. We used the inferred *C* to characterize coupling mechanisms in the network, which contained information on coupling strength and direction. The coupling strength is used to quantify the coupling amplitude, the Euclidean norm of inferred coupling coefficient matrix from oscillators ϕ_*i*_ and ϕ_*j*_.

In this study, we established coupling functions based on coupled-phase-oscillator model in a pairwise manner and utilized Dynamical Bayesian theory to infer directed coupling interactions for all possible pairs of channels. For the two channels, *i* and *j*, we calculated the coupling influence that *i* exerted on *j*(*c*_*i*→*j*_) and *j* on *i*(*c*_*j*→*i*_) in each frequency interval and posture state of each subject. Meanwhile, the value of *c*_*i*→*j*_ is defined as the coupling strength from channel *i* to *j*. A total of 1,260 frequency-specific directed coupling interactions were derived from all possible pairs of 36 channel signals for each subject and specific posture state. We used surrogate data testing to validate coupling interaction results. A total of 100 amplitude-adjusted Fourier transform (AAFT) surrogate signals were generated for every channel by shuffling the phases of the original time series to create new time series with the same means, variances, and autocorrelation functions as the original sequences but without their phase relations (Bernjak et al., 2012; Tan et al., 2016). The directed coupling interactions were derived from all possible pairs of the 100 surrogate signals for every two channels based on coupled-phase-oscillator model and dynamical Bayesian theory. We obtained the average of all coupling measurements calculated from these surrogate realizations of the signals. The directed coupling strength of the experimental signals was considered significant if the value was 2 standard deviations (SD) above the surrogate means. The coupling strength from the test denoted significant interaction. Results of the directed coupling interaction include coupling strength and direction. EC is derived from the concept of directed coupling interaction, which was based on a specific model of causal dynamics, to determine the causal influence that one oscillator exerts on another (Park and Friston, 2013). For each subject, the coupling interactions among all possible pairs of 36 channels were computed and tested by surrogate measurements for the specific posture state and frequency intervals.

#### Posture-dependent group-averaged EC network

To construct the EC network at group level, we must test the existence of a connection between channels. Based on the individual significant connectivity, a connection at group level was considered when the connections between two channels exist in over 75% of the subjects. For group EC, the strength of each connection was the average coupling strength between two channels. The significant interactions of EC network are shown for each group and posture state.

#### Main coupling direction among the six brain regions

When the value of *C*_*i*→*j*_ exceeded *C*_*j*→*i*_, we defined *i*→*j* as the main coupling direction (mCD) between oscillator ϕ_*i*_ and oscillator ϕ_*j*_. For any individual subject, every significant interaction of all possible pairs of channels between two brain regions includes a mCD, either from region 1 to region 2 or from region 2 to region 1. Binomial tests were performed on the mCDs of significant interactions between each two brain regions to determine significant difference. A significant difference indicated the presence of mCD, which plays a dominant role in the coupling function between the two brain regions; otherwise, bidirectional coupling between the two brain regions was considered.

#### EC among the six brain regions

This paper aimed to analyze the interaction among brain regions. For each subject, the coupling strengths of significant interactions between all possible pairs of 36 channels were averaged as over $A_{6}^{2}=30$ directed interregional connection types, thus corresponding to mutual interactions among the six regions. The channel distributions covered by each brain region are displayed in Figure 1. The 30 EC values were obtained from each subject by averaging the coupling strength of channels between two brain regions for specific frequency interval and posture state.

#### Correlation analysis between interregional EC and cognitive performance

To identify the association between the varying causal interactions and the cognitive performance of the elderly, we extracted and correlated the interregional coupling strength with the MMSE scores using Pearson's correlative analysis.

### Statistical analysis

Analysis of significant differences in the characteristics between young and elderly subjects was conducted using *t*-test for means and SD. Chi-square test was performed to evaluate the significant difference in sex percentages. Binomial-tests were performed on the mCDs of significant channel pairs between the two brain regions to determine the coupling direction of the inter-regions. Given the non-normal distribution of data, we conducted Kruskal–Wallis to test the significance level of frequency-specific EC of 30 connection types among different groups and conditions. The association between the interregional EC and cognitive performance was explored using Pearson correlation coefficients. A difference of *p* < 0.05 was considered statistically significant.

## Results

### Demographic data

Participant characteristics are shown in Table 1. The two groups has similar body mass index (BMI) nut significantly different MMSE scores (*p* < 0.0001).

### Posture-related changes in EC network

**Figure 2:**
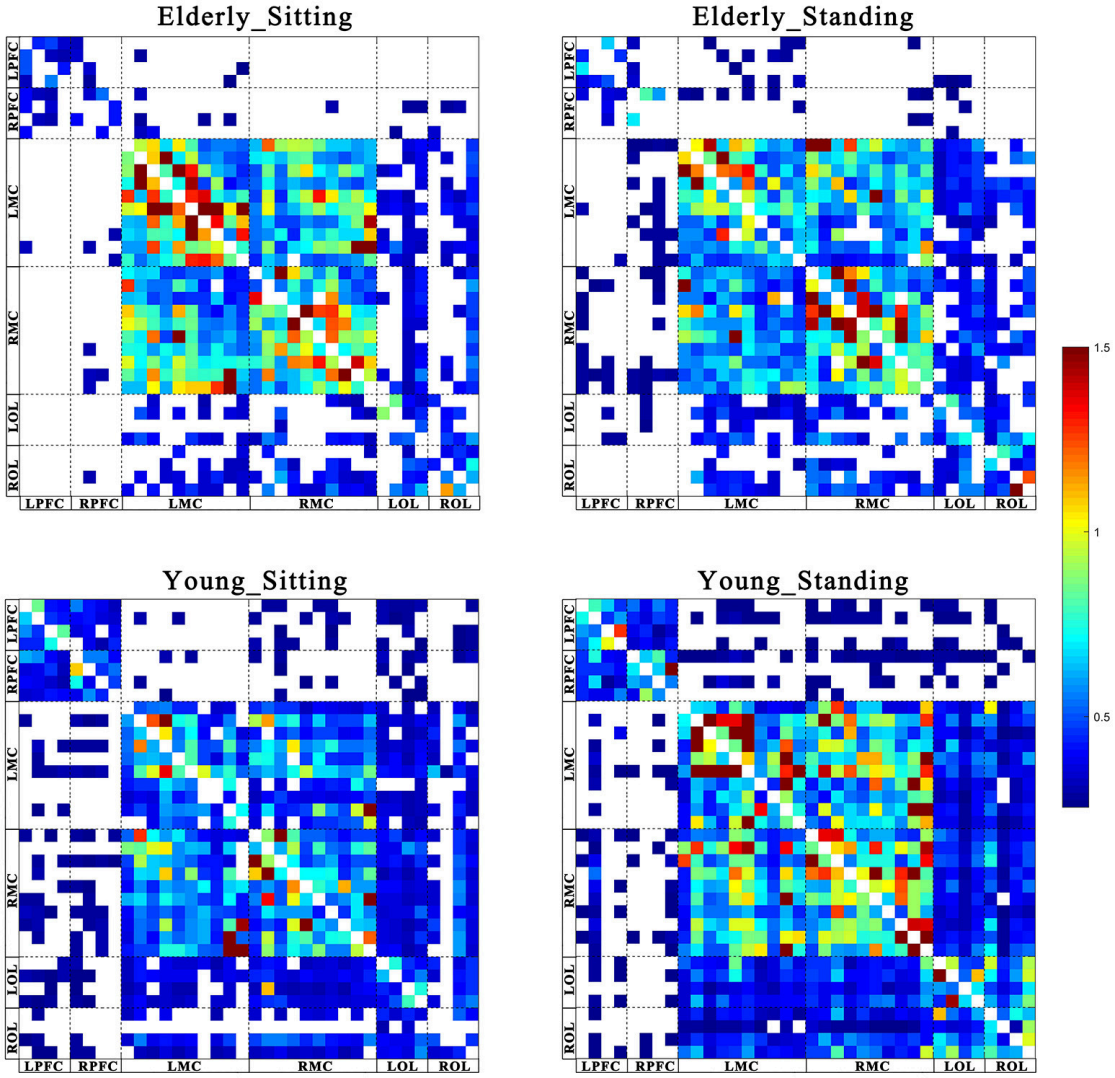
Posture-related changes in effective connectivity network in frequency interval I. The frequency-specific asymmetric mean coupling strength matrix of two groups in different conditions (where 36 is the number of channels). The coupling direction between the channels is indicated from the row to the column. Different channels correspond to brain regions, Channel 1–4 (LPFC), Channel 5–8 (RPFC), Channel 9–18(LMC), Channel 19–28 (RMC), Channel 29–32 (LOL), and Channel 33–36 (ROL).

**Figure 3:**
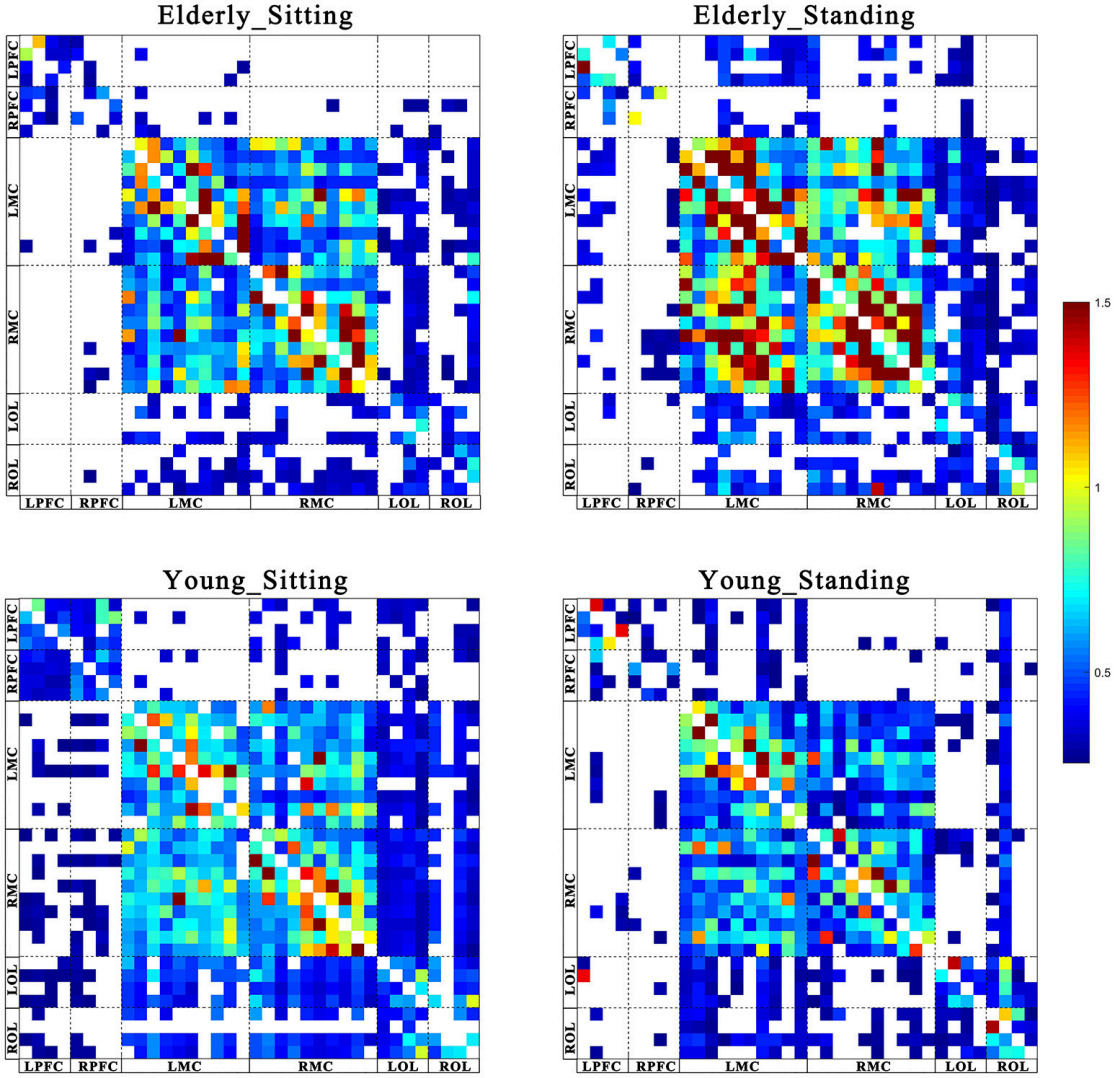
Posture-related changes in effective connectivity network in frequency interval II. The frequency-specific asymmetric mean coupling strength matrix of two groups in different conditions (where 36 is the number of channels). The coupling direction between the channels is indicated from the row to the column. Different channels correspond to brain regions, Channel 1–4 (LPFC), Channel 5–8 (RPFC), Channel 9–18(LMC), Channel 19–28 (RMC), Channel 29–32 (LOL), and Channel 33–36 (ROL).

Figures 2, 3 show the EC network of the 36 channels at the group level for specific frequency interval and posture states of the elderly group and young group. Different channel distributions represented different brain regions as displayed in the diagrams. Posture-related changes were observed in the EC network in different frequency intervals. A matrix box with color indicates a significant EC value between two channels, whereas the white matrix lattice represents no significant connection. Different colors represent different values of coupling strength.

### Age-related changes in mCD between the brain regions

In this study, we calculated the interregional EC among the six brain regions: LPFC, RPFC, LMC, RMC, LOL, and ROL. The mCDs among the brain regions are either bidirectional or unidirectional. Bidirectional connection between the two brain regions illustrates absence of dominant functional source of coupling. Thus, the two brain regions regulate each other. On the contrary, unidirectional connection indicates that one of the two brain regions serves as the predominant functional source of coupling and plays a major regulatory role, whereas the other region acts as the target of regulation. The reverse of the mCD is regarded as the feedback effect in this study.

**Figure 4:**
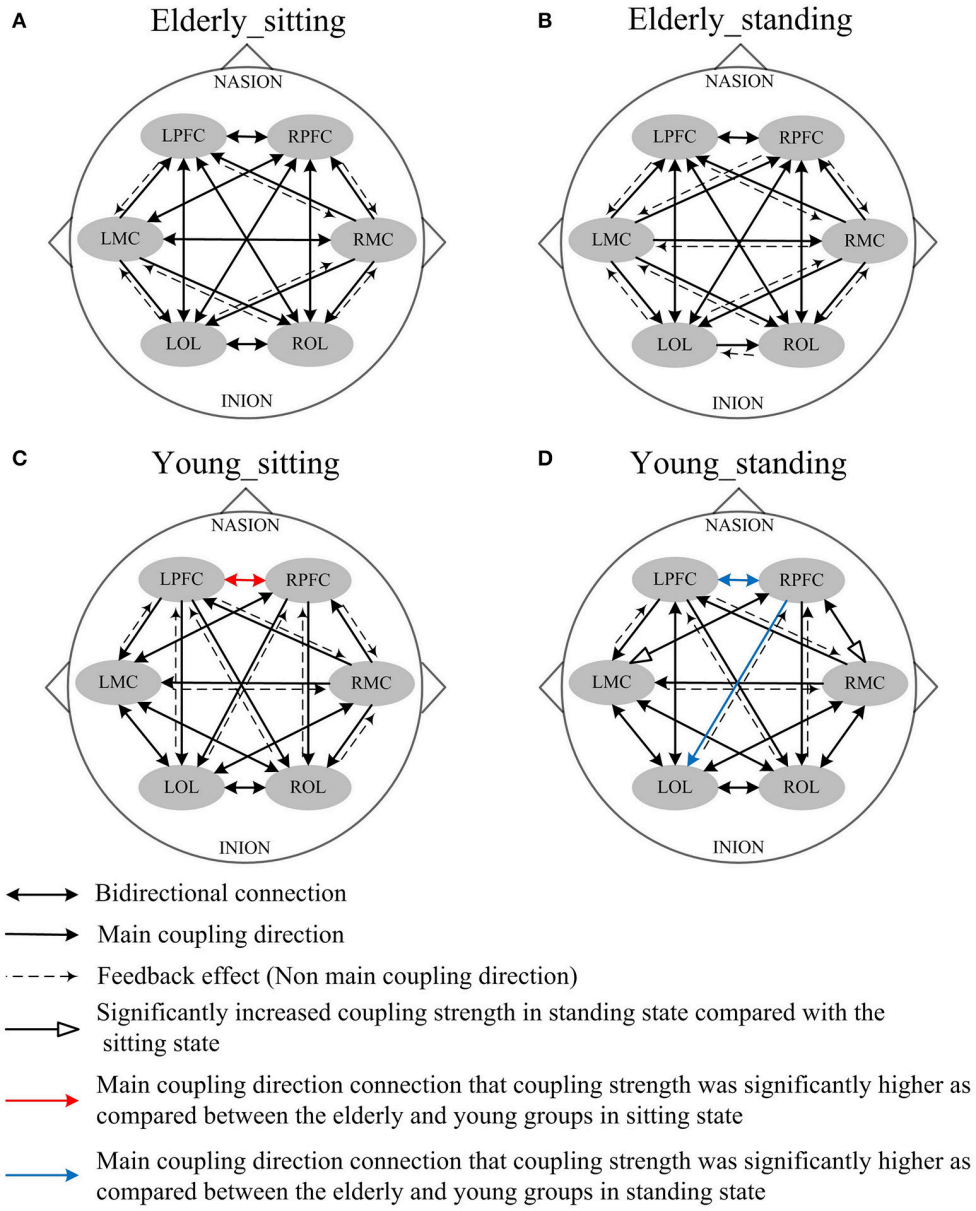
Comparison of the main coupling direction in interval I in the effective connectivity network among the six brain regions. The arrow points to the direction of the coupling interaction. Unidirectional arrows and bidirectional arrows are present between the two brain regions. **(A)** Elderly_sitting state, **(B)** Elderly_standing state, **(C)** Young_sitting state, **(D)** Young_standing state.

**Figure 5:**
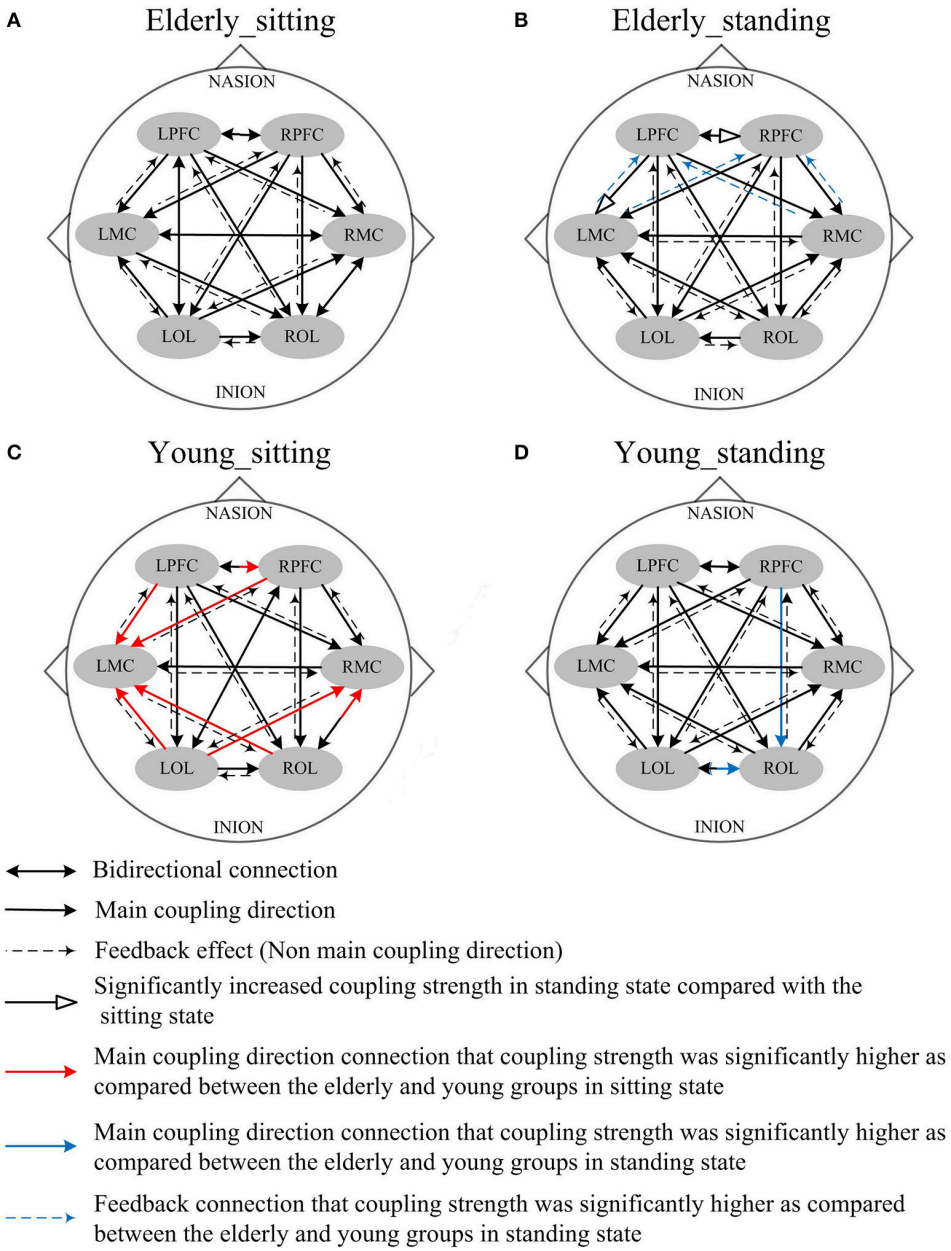
Comparison of the main coupling direction in interval II in the effective connectivity network among the six brain regions. The arrow points to the direction of the coupling interaction. Unidirectional arrows and bidirectional arrows are present between the two brain regions. **(A)** Elderly_sitting state, **(B)** Elderly_standing state, **(C)** Young_sitting state, **(D)** Young_standing state.

Figures 4, 5 illustrate the mCD among the six brain regions in both groups and conditions. In interval I (Figure 4), from sitting to standing states, the coupling direction between the LMC and RPFC, LMC and RMC, and LOL and ROL in the elderly was transformed from bidirectional to unidirectional connection. This finding implies that MC played a stronger regulatory role in the standing state among the regions of the brain. In the young group, posture-related changes in mCD illustrated the regulation exerted by RPFC and ROL on RMC. This effect was increased in the standing state. The most predominant finding in this interval includes age-related changes in mCD among the brain regions. The mCD in the elderly was mostly observed from MC to PFC and to OL, indicating that MC played a main regulatory role in the brain network of the elderly. However, the connections between PFC and OL were bidirectional in the elderly but unidirectional in the young group (from PFC to OL). In summary, these results illustrated the occurrence of age-related deterioration in the regulation function of PFC, especially for long-distance connections. Nonetheless, the MC in the elderly group plays a major regulatory role to compensate for the decline regulation function of PFC.

Figure 5 shows the posture-related changes in the mCD in interval II. In general, regulation from OL to MC and from PFC to OL increased in response to posture changes in both groups. In the sitting state, the mCD is from LMC to ROL in the elderly but from ROL to LMC in the young group.

### Age-related changes in EC under different posture states

**Figure 6:**
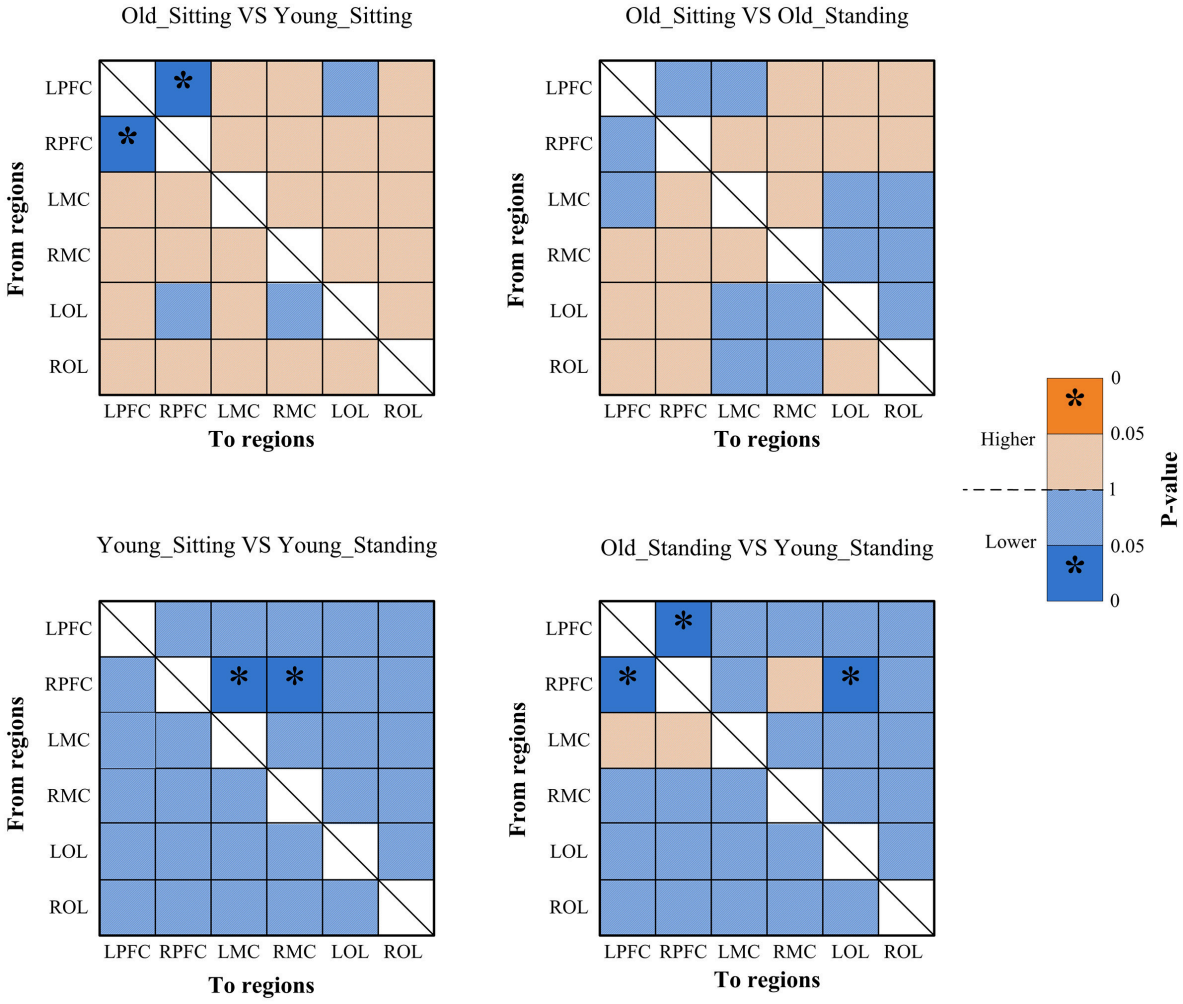
Significance matrices representing significant difference between each two groups in EC among the six brain regions in interval I. In every two groups of contrast, the warm color represents that the former is greater than the latter, and the bright color with ^*^represents a significance that is greater than the latter. Cold color represents that the former is less than the latter, and the darker cold color with ^*^represents significance lower than the latter.

**Figure 7:**
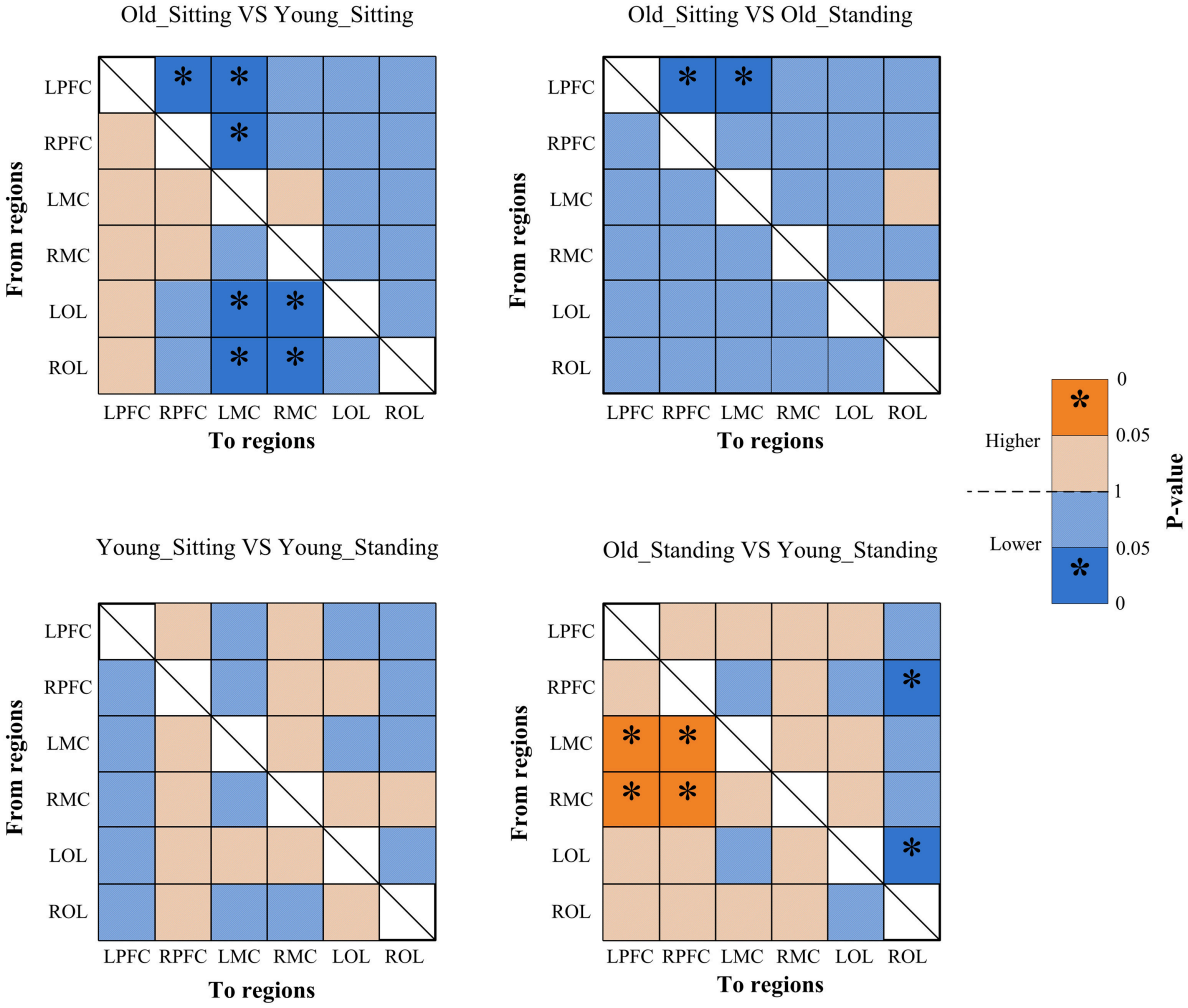
Significance matrices representing significant difference between each two groups in EC among the six brain regions in interval II. In every two groups of contrast, the warm color represents that the former is greater than the latter, and the bright color with ^*^represents a significance greater than the latter. Cold color represents that the former is less than the latter, and the darker cold color with ^*^represents significance lower than the latter.

Coupling strengths of the significant connection between channel pairs were averaged to form 30 directed interregional EC types among the six brain regions. Frequency-specific connectivity was analyzed in both groups and both posture conditions to reveal the age-related alterations in EC from sitting to standing states. Figures 6, 7 show the significant changes in EC values among the six brain regions in both groups and conditions.

#### Posture-related changes in EC

From sitting to standing state, the coupling strengths of RPFC to LMC (*p* = 0.03) and RPFC to RMC (*p* = 0.02) (represented by RPFC→ LMC, RPFC→ RMC) were significantly increased in interval I (Figure 6) of the young group. By contrast, the coupling strength significantly increased from LPFC to RPFC (*p* = 0.008) and from LPFC to LMC (*p* = 0.031) (represented by LPFC→RPFC, LPFC→LMC) in interval II (Figure 7) of the elderly group.

#### Age-related changes in EC

Compared with the young group, in the sitting state, the elderly group had significantly decreased EC as noted in LPFC→RPFC (*p* = 0.023) and RPFC→LPFC (*p* = 0.044) in interval I (Figure 6); and LPFC→RPFC (*p* = 0.028), LPFC→LMC (*p* = 0.02), RPFC→LMC (*p* = 0.042), LOL→LMC (*p* = 0.004), ROL→LMC (*p* = 0.014), ROL→RMC (*p* = 0.012), and ROL→RMC (*p* = 0.022) in interval II (Figure 7). Compared with the young group, the elderly group in the standing state had significantly increased EC of LMC→LPFC (*p* = 0.023), LMC→RPFC (*p* = 0.025), RMC→LPFC (*p* = 0.022), and RMC→RPFC (*p* = 0.009) in interval II (Figure 7). According to the results of mCD among the brain regions, the connections from MC to PFC in interval II of the elderly group in standing state had significantly higher coupling strength than those of the young group. This finding corresponded to the feedback connections in the elderly group.

#### Correlations between interregional EC and cognitive performance

We found that the interregional causal interactions were correlated with MMSE scores in the elderly (Table 2). The MMSE scores were significantly and positively related with the directed coupling strength of LPFC→RPFC (*r* = 0.689, *p* = 0.002), RPFC→LPFC (*r* = 0.679, *p* = 0.003), RPFC→LMC (*r* = 0.487, *p* = 0.047), LMC→RPFC (*r* = 0.486, *p* = 0.048), RPFC→RMC (*r* = 0.531, *p* = 0.028), RPFC→LOL (*r* = 0.505, *p* = 0.039), RPFC→ROL (*r* = 0.489, *p* = 0.047) in interval I.

**Table 2:** Correlations between interregional EC and cognitive performance.

**State**	**Interval**	**Interregional EC**	**MMSE scores**	
			***R***	***P***
Sitting	I	LPFC→RPFC	0.689	0.002^*^
		RPFC→LPFC	0.679	0.003^*^
		RPFC→LMC	0.487	0.047^*^
		LMC→RPFC	0.486	0.048^*^
		RPFC→RMC	0.531	0.028^*^
		RPFC→LOL	0.505	0.039^*^
		RPFC→ROL	0.489	0.047^*^

* *Denotes the significant correlations, p < 0.05*.

## Discussion

NIRS signals recorded on the head consist of endogenous systemic activity and neurovascular coupling (Scholkmann et al., 2014). Oscillations in NIRS at different frequency intervals may reflect different neurovascular couplings and systemic regulation mechanisms. These regulatory mechanisms are considered working together to maintain a relatively stable rCBF at different states. The main findings of this study are the age-related alterations in EC in response to posture changes. From sitting to standing state, the influence of RPFC on MC significantly increased in interval I for the young group. However, the effect of LPFC on RPFC and LMC significantly increased in interval II for the elderly group. Compared with the young group, the elderly subjects had significantly decreased interregional coupling strength in intervals I and II in their sitting state. The decreased connectivity between LPFC and RPFC in interval I was also positively correlated with MMSE scores of the elderly subjects. The EC from MC to PFC in interval II was increased in elderly subjects in standing state, as compared with that in the young subjects. These findings revealed the age-related changes in reorganization of interregional interactions under different posture states and highlight the alterations of healthy aging in neurovascular coupling functions. These results may provide new insights into the development of new strategies to monitor cognitive loss and detect the functional performance of neurovascular coupling mechanism in the elderly.

The brain network is a coordinated system of specific brain regions working together to promote specific cognitive functions (Andrewshanna et al., 2007; Meunier et al., 2009; Wen et al., 2011). Structural, functional, and directional organizations of complex brain networks serve as basis for the realization of various brain functions. The interactions among the brain regions are different in various posture states. In this study, EC was calculated from neurovascular coupling signals based on a model of coupled phase oscillators and Dynamical Bayesian inference. The findings enabled us to estimate the coupling strength, mCD and underlying causality among brain regions in the sitting and standing states. Age-related alterations in the brain network in different posture states were analyzed according to the physiological meanings of oscillation signals in different frequency intervals (Li et al., 2013a; Han et al., 2014; Bu et al., 2016).

The results showed significant age-related differences in EC in response to posture changes. Coupling strength analysis in interval II (Figure 7) indicated that the EC in elderly was significantly affected by posture. However, the same result was not observed in the young group. Within the brain, the cerebral oscillations in interval II were suggested as locally neurogenic activities originating from vascular reactions of neurogenic origin (Shiogai et al., 2010; Li et al., 2013b). Brain vasculature can adjust blood flow in response to local conditions. Neurogenic control of cerebral blood vessels is innervated by the surrounding vasoactive nerves, that is., sympathetic, parasympathetic and somatic sensory nerves on the brain surface, and is sufficient for regulating overall blood flow to the brain (Hotta, 2016). Sustained activity of the autonomous nervous system maintains the basal level of blood vessel contraction by releasing substances that affect smooth muscle activity. Hemodynamic parameters are closely regulated through tight neurogenic innervations and under partially autonomic control in interval II (Zhang et al., 2002; Shiogai et al., 2010). In the elderly group, causal influence from LPFC to RPFC and to LMC was significantly stronger in the standing state than that in the sitting state. A study based on EEG showed that the theta activities in fronto-central and centro-parietal cortical areas significantly increased with increasing balance task demands. The dynamic reorganization of the cortical network can contribute to an optimization of balance control (Mierau et al., 2015, 2017). According to the mCD results among the brain regions, the causal influences from OL to MC and from PFC to OL were more dominant during standing than during sitting. Different postures lead to differences in hemodynamic responses in the cerebral and systemic vasculature, where autonomic reflexes are mediated by sympathetic, parasympathetic, and somatic sensory nerves (Olufsen et al., 2004). Pfeifer reported the decreased functions of sympathetic nervous system with aging (Pfeifer et al., 1983). The significant difference in EC between sitting and standing states in the elderly revealed that reorganization of the interregional interactions is required to maintain the performance in different posture states.

Compared with the young group in sitting state, the elderly group generally decreased directed interactions among the six brain regions. Our study revealed that the elderly exhibited significantly decreased regulating influence from PFC and OL to MC. Age-related decrease in EC across brain regions suggested decreased regulation on motor area in the elderly. This phenomenon may be due to the significantly attenuated function of neurovascular coupling control in PFC and OL with aging. This conclusion is supported by the finding that age-related shrinkage in gray matter volume and deterioration in cerebral white matter integrity are mainly distributed in frontal areas (Park and Reuterlorenz, 2009). These results may also be related to the connectivity between frontal–parietal areas, which are impaired in aging as shown from structural imaging (Burzynska et al., 2010; Meier-Ruge et al., 2010; Madden et al., 2012). A previous study reported that the disturbance and misperception of the body schema was caused by lesions in the OL (Coslett, 1998). Basing on our results, we suggest that the attenuated causal influence from OL to MC explains why elderly people fall easily. In interval II, the results in standing state showed significantly increased coupling strength from MC to PFC in elderly subjects as compared with that in the young group. That is, the elderly exerted great influence from MC to PFC during standing state. This result indicates that the elderly greatly relies on the PFC and its role in cognitive resources during standing state. This finding is consistent with the results of previous studies that additional cognitive resources need to be recruited as a measure of compensation to maintain performance with aging (Vandenbossche et al., 2011; Beurskens et al., 2014). By combining this finding and the mCD results across the brain network (Figure 5), we observed that the significantly increased causal interactions from MC to PFC in the elderly are a feedback effect. The mCD in this interval was approximately consistent in both groups. PFC played a predominant regulatory role on OL and MC and MC was also regulated by the OL.

The results also revealed the existence of age-related changes in EC in response to posture changes in interval I. The origin of the oscillations in interval I (0.052–0.145 Hz) is associated with vasomotor activities, thus contributing to the regulation of rCBF according to regional metabolic demands. The realization of brain function is critically dependent on a steady supply of blood (Girouard and Iadecola, 2006; Shiogai et al., 2010). With an increase or decrease in intravascular pressure, the vascular smooth muscles contract or relax to correspond with the blood oxygen concentration (Shiogai et al., 2010). The cerebral oscillations originated locally from intrinsic myogenic activity of smooth vascular muscle cells and were endowed with neural autonomic control of cerebral circulation to assure cerebrovascular function (Rowley et al., 2007; Shiogai et al., 2010). Under different conditions, myogenic regulatory mechanism plays a certain role in stabilizing CBF fluctuation. By comparing the effective interactions among the network in different posture states, we observed that the coupling strength from RPFC to MC in interval I significantly increased in the young group in response to posture changes. The elderly group exhibited no significant difference in EC during sitting and standing states. These results revealed that according to varying postures, different regional metabolic demands are required to meet stable CBF. This phenomenon may result in differences in the interaction among brain regions. The causal influence from PFC to MC in the young group was significantly stronger in the standing state than in the sitting state. These regions were assumed to play key roles in cognitive control function (Miller and Cohen, 2001) and coordination of sensory and motor functions (Basso et al., 2014). These results may be attributed to the strong myogenic regulation mechanism in the young group and result in prompt and reliable regulation of rCBF in response to posture changes. The absence of significant difference in response to posture change in the elderly may be due to the increased vessel stiffness with aging and the myogenic regulation degeneration in the elderly group.

In the sitting state, the directed coupling strength between LPFC and RPFC was significantly lower in the elderly group compared with the young group, indicating that mutual causal interaction between them decreased with aging. Such decreased connectivity was significantly correlated with the MMSE scores in elderly, which indicated the cognitive performance. PFC plays a key role in complex cognitive behavior (Miller and Cohen, 2001). Based on the results of the positive relationship between the cognitive performance and causal interactions between left PFC and right PFC, we speculated that such decreased EC may be an indicator of cognitive decline. This finding was in line with the idea that the altered patterns of connectivity between PFC and other regions attributed to the impairment in cognitive control (Meunier et al., 2009). These results agree with those of previous studies, showing that frontal functions are susceptible to degenerative changes in normal aging (Park and Reuterlorenz, 2009; Antonenko and Flöel, 2013). These results may be attributed to reduced spontaneous activity in microvascular smooth muscle cells together with increased vessel stiffness with aging (Schroeter et al., 2004; Li et al., 2013a). Other notable findings in this interval were the age-related changes in mCD among the brain regions. In the young group, PFC predominantly influenced MC and OL. However, in the elderly group, the mCD results showed that the MC predominantly influenced PFC and OL, implying that MC played a main regulatory role in interval I. By combing present and previous results on coupling strength, we revealed the impaired prefrontal regulation in the elderly and the theory that long-ranged regulatory mechanisms become vulnerable with aging (Dardo and Volkow, 2012). In the elderly group, the regulating ability of smooth muscle cells in MC was preserved or enhanced in interval I. The brain possibly reorganizes the regulation ability of smooth muscle cells to adapt to decreased cognitive deficits with age.

## Limitations

One consideration of the results is that systemic activity interferences in this interval must be further addressed. In our study, the range of interval I was within 0.052–0. 145 Hz. We cannot fully exclude contamination by heart rate and blood pressure fluctuations. Cerebral oscillations in interval I were considered to originate locally from the intrinsic myogenic activity of smooth vascular muscle cells and partly under neural autonomic control of cerebral circulation (Rowley et al., 2007; Shiogai et al., 2010; Li et al., 2012). A study reported that in the frequency interval 0.04–0.15 Hz, the contribution of systemic signals to hemodynamic changes reached only 35% when carried by oxy-Hb (Katura et al., 2006). These systemic activity interferences may affect the results of EC. Thus, the interference of Mayer waves should be considered in future studies.

## Conclusions

In this study, we revealed posture-related changes in frequency-specific EC in elderly subjects based on dynamic Bayesian inference. The brain network features different regulatory modes at different frequencies, which change to maintain stable CBF in different posture states. The results reflect the age-related changes in effective interactions across brain regions in response to posture changes. Significantly decreased interregional EC was found in intervals I and II in elderly in the sitting state as compared with that in the young group. The decreased connectivity in interval I was also strongly positively correlated with cognitive performance in the elderly. Compared with that in the young group, the influence from MC to PFC significantly increased in intervals II in the elderly group in standing state, suggesting that the elderly subjects greatly rely on cognitive resources as a compensation to maintain performance in aging. These findings may provide an evidence for cognitive decline and can aid in detecting the functional performance of neurovascular coupling mechanism in the elderly.

## Author contributions

ZL: designed the study and edited the manuscript; CH: did the experiment, analyzed the data and drafted the manuscript; LB: did the experiment; GX: analyzed the data; YL: performed the statistical analysis; MZ: contributed to the physiological interpretation of the results; LS: edited the manuscript.

### Conflict of interest statement

The authors declare that the research was conducted in the absence of any commercial or financial relationships that could be construed as a potential conflict of interest.

## Abbreviations

EC: Effective Connectivity
FC: Functional Connectivity
LPFC: Left Prefrontal Cortex
RPFC: Right Prefrontal Cortex
LMC: Left Motor Cortex
RMC: Right Motor Cortex
LOL: Left Occipital Lobe
ROL: Right Occipital Lobe
WT: Wavelet Transform
mCD: main coupling direction.
